# Global Chromatin Architecture Reflects Pluripotency and Lineage Commitment in the Early Mouse Embryo

**DOI:** 10.1371/journal.pone.0010531

**Published:** 2010-05-07

**Authors:** Kashif Ahmed, Hesam Dehghani, Peter Rugg-Gunn, Eden Fussner, Janet Rossant, David P. Bazett-Jones

**Affiliations:** 1 Genetics and Genome Biology Program, The Hospital for Sick Children, Toronto, Ontario, Canada; 2 Department of Physiology, School of Veterinary Medicine and Institute of Biotechnology, Ferdowsi University of Mashhad, Mashhad, Iran; 3 Developmental and Stem Cell Biology Program, The Hospital for Sick Children, Toronto, Ontario, Canada; Ludwig-Maximilians-Universität München, Germany

## Abstract

An open chromatin architecture devoid of compact chromatin is thought to be associated with pluripotency in embryonic stem cells. Establishing this distinct epigenetic state may also be required for somatic cell reprogramming. However, there has been little direct examination of global structural domains of chromatin during the founding and loss of pluripotency that occurs in preimplantation mouse development. Here, we used electron spectroscopic imaging to examine large-scale chromatin structural changes during the transition from one-cell to early postimplantation stage embryos. In one-cell embryos chromatin was extensively dispersed with no noticeable accumulation at the nuclear envelope. Major changes were observed from one-cell to two-cell stage embryos, where chromatin became confined to discrete blocks of compaction and with an increased concentration at the nuclear envelope. In eight-cell embryos and pluripotent epiblast cells, chromatin was primarily distributed as an extended meshwork of uncompacted fibres and was indistinguishable from chromatin organization in embryonic stem cells. In contrast, lineage-committed trophectoderm and primitive endoderm cells, and the stem cell lines derived from these tissues, displayed higher levels of chromatin compaction, suggesting an association between developmental potential and chromatin organisation. We examined this association *in vivo* and found that deletion of *Oct4*, a factor required for pluripotency, caused the formation of large blocks of compact chromatin in putative epiblast cells. Together, these studies show that an open chromatin architecture is established in the embryonic lineages during development and is sufficient to distinguish pluripotent cells from tissue-restricted progenitor cells.

## Introduction

The initial steps of mouse preimplantation development involve an ordered series of cleavage divisions to form an 8-cell embryo. Cell fate decisions then allocate cells to the embryonic or extra-embryonic lineages and by the time of embryo implantation three distinct cell populations have been established [1]. Trophectoderm (TE) and primitive endoderm (PE) cells generate the extra-embryonic tissues, whereas epiblast (EPI) cells are pluripotent and give rise to the embryo itself. When EPI cells are removed from the embryo and explanted into culture they give rise to embryonic stem (ES) cells, which retain epiblast identity and potency [2].

The genome of eukaryotic cells is organised into euchromatin, which is generally permissive for gene activation, and heterochromatin, which is largely gene-poor or transcriptionally silenced. It is presumed that chromatin compaction can influence transcriptional activities by regulating accessibility to transcription factors and DNA interactions [3]–[5]. Similarly, the location of the chromosome within the nucleus is thought to control gene activity, with the nuclear periphery associated with transcriptionally repressed chromatin [6]–[9]. Nuclear architecture can be altered by a number of distinct pathways including ATP-dependent nucleosome remodelling, linker histone proteins, histone variants and covalent modifications to the histones and DNA [10].

Although the chromatin structure of ES cells has been extensively analysed, there has been little comparison to the equivalent cells within the embryo itself. ES cell chromatin exists in an unusual configuration with widely dispersed open chromatin [11]. Following differentiation, there is extensive reorganisation and large compact chromatin domains are formed [11], [12]. These structural changes correlate with a reduction in transcriptional activity and rate of histone protein exchange that also occurs upon differentiation [11], [13]. These observations have led to the idea that an open chromatin structure may be required for stem cell pluripotency. Consistent with this proposal, reduced expression of numerous chromatin-remodelling proteins in ES cells result in an accumulation of compact chromatin, disruption to self-renewal and altered ES cell differentiation potential [14]–[19]. These studies suggest that a dispersed chromatin architecture may contribute to stem cell behaviour in general. However, it is unclear whether this epigenetic state truly reflects the status of pluripotent cells in the embryo itself or if it is acquired during the selection for self-renewal *in vitro*. It is essential, therefore, to examine the global chromatin architecture in pluripotent cells from the early mouse embryo.

It is also important to understand how a dispersed chromatin state is formed during the establishment of pluripotency. Knockdown of the Chd1 remodelling protein causes a decrease in somatic cell reprogramming efficiency [15], suggesting that acquiring pluripotency is dependent on chromatin remodelling activity. Similarly, the formation of a functional zygotic nucleus following fertilization, and the subsequent development from a one-cell to an eight-cell embryo, also requires extensive alterations in chromatin structure that may be linked to developmental potential. During these stages in development there is deposition of linker histones and high-mobility group proteins onto chromatin [20]–[26], exchange of histone variants [27], [28] and changes to DNA methylation levels [29]–[33], which suggest that remodelling of chromatin structure is important for directing differentiated gamete pronuclei into a pluripotent state. Although these studies were critical to our understanding of reprogramming events, they all examined indirect indicators of chromatin structure and so it remains important to directly visualize the chromatin architecture during preimplantation mouse development.

To these ends, we used electron spectroscopic imaging (ESI) to observe directly the ultrastructural changes in chromatin during the transition from one-cell to early postimplantation stage embryos. This technique enabled us to visualize chromatin structure during the establishment of pluripotency that occurs between fertilization and the emergence of epiblast cells, and also in the subsequent restriction of cell potential that occurs during lineage allocation. We found that blocks of compact chromatin formed between one-cell and two-cell embryos, but successive stages of development were associated with chromatin decondensation. Strikingly, the chromatin organization in early epiblast cells was indistinguishable from the highly dispersed chromatin seen in ES cells. Two observations revealed that this distinct epigenetic state was dependent on developmental potential. First, lineage-committed TE and PE cells, and the stem cell lines derived from these tissues, revealed higher levels of compact chromatin formation. Second, zygotic deletion of the key pluripotency factor *Oct4* caused epiblast cells to undergo extensive chromatin compaction. Together, these studies show that an open chromatin architecture is established in the embryonic lineages during development and is sufficient to distinguish pluripotent cells from tissue-restricted progenitor cells.

## Results

We used ESI to observe directly the ultrastructural changes in chromatin during the transition from one-cell to early postimplantation stage embryos. This imaging technique generates nitrogen and phosphorus maps that can be used to distinguish chromatin, ribonucleoproteins (RNPs) and protein-based structures [34]–[39]. In addition, the high-contrast phosphorus mapping enables detailed chromatin structures to be visualized, including the presence of nucleosomes, fibre-fibre distances and the detection of linker DNA. For all pre-implantation stages, we have also included a corresponding image with the fluorochrome 4,6-diamidino-2-phenylindole (DAPI). This DNA counter-stain preferentially binds to AT-rich major satellite sequences and is able to reveal variations in DNA density but does not provide an accurate indication of chromatin compaction levels [37].

### Compaction of chromatin between one-cell and two-cell stage embryos

**Figure 1:**
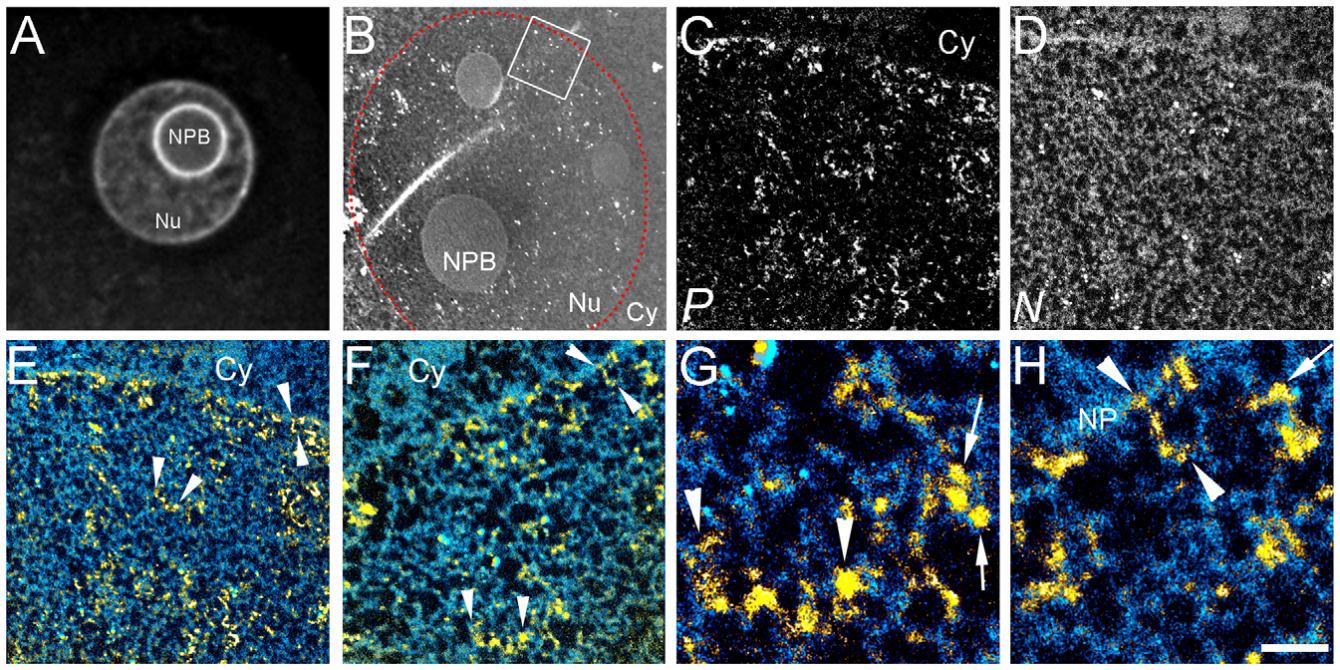
Highly dispersed 10 nm chromatin fibres in pronuclei of one-cell embryo. (A) Fluorescence image of a DAPI stained male pronucleus. (B) Low magnification mass image of a male pronucleus (nuclear periphery highlighted with dotted line; Nu, nucleus; Cy, cytoplasm; NPB, nucleolar precursor body; NP, nuclear pore). Phosphorus (*P*) and nitrogen (*N*) maps of the indicated field are shown in C and D respectively. Relationships of nucleic acid- (yellow) and protein-based (blue) structures of the field in C, D is shown at two magnifications in E and F. Phosphorus signal (yellow) is merged with the nitrogen minus the phosphorus map (see Materials and Methods). Chromatin fibres indicated (E,F) (arrowheads) are shown at higher magnification (G,H, respectively). Nucleosomes, identified on the basis of dimensions and phosphorus and nitrogen content are indicated (arrows, G,H). Scale bar in H represents 7 µm (A), 3 µm (B), 500 nm (C–E), 250 nm (F), and 60 nm (G,H).

**Figure 2:**
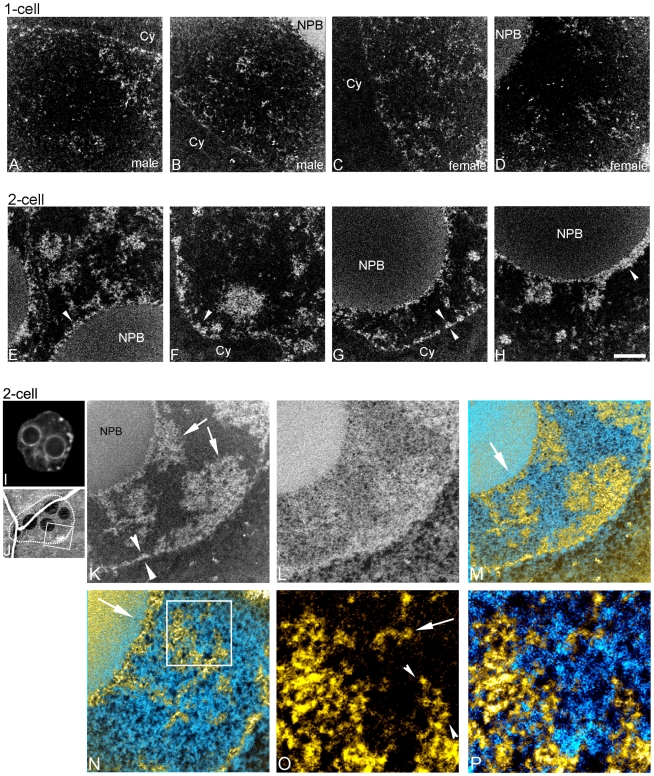
Chromatin becomes more compact and concentrated at nuclear envelope and periphery of nucleolar precursor bodies between one-and two-cell stage embryos. Phosphorus maps (white on black background) of male (A,B) and female (C,D) pronuclei in one-cell embryo. Phosphorus maps of nuclei of two-cell embryos (E–H) (NPB, nucleolar precursor body; Cy, cytoplasm). Fluorescence image of a DAPI stained nucleus of a two-cell embryo (I). Low magnification mass image (J) of two-cell embryo showing the nucleus imaged in K–P. Phosphorus (K) and nitrogen (L) maps of a field of the two-cell embryo. Arrowheads (K) indicate chromatin organization along nuclear envelope, and arrows (K) indicate blocks of compact chromatin. Merge of phosphorus and nitrogen maps (M). N, higher magnification of field from M. Arrow shows relative orientation of the lower (M) and higher magnification view (N), and indicates chromatin accumulation on the periphery of a NPB. Phosphorus map (O) and merged phosphorus and nitrogen maps (P) of boxed region (N) are shown at higher magnification. Loop(s) of 10 nm chromatin fibre on the edge of a block of compact chromatin is indicated with arrowheads, and a chromatin fibre is indicated with arrow (O). Scale bar in H represents 500 nm (A–H), 7.5 µm (I), 400 nm (K–M), 250 nm (N) and 80 nm (O,P).

We examined the chromatin and nuclear architecture of male and female pronuclei in the one-cell embryo (nine hours after hCG administration). At this developmental stage, the structural features at low (Figure 1A,B) or high magnification (Figure 1C–F) were identical between male and female pronuclei (compare Figure 2A,B to C,D). Dispersed and highly folded 10 nm chromatin fibres, the lowest level of chromatin organization, were visualized throughout the nucleoplasm. The arrowheads in the highest magnification images (Figure 1G,H) indicate short segments of 10 nm chromatin fibres. The arrows indicate structures that are consistent with nucleosomes, based on their dimensions and on their phosphorus to nitrogen ratio [36]. Whereas the nuclear envelope and nucleolar periphery are sites of chromatin compaction in most differentiated cell types, these regions in one-cell embryo nuclei did not display significant chromatin accumulation (Figure 1C and Figure 2A–D). The AT-richness of the repetitive DNA, that is reported to accumulate at the nucleolar precursor body (NPB) surface [40], [41], may give an exaggerated impression of the amount of DNA in this region (Figure 1A). Regardless, unlike the mature nucleolus seen at later stages and in almost all differentiated cell types examined by conventional transmission electron microscopy, the NPBs at this stage displayed a low propensity to concentrate chromatin fibres (Figure 2B,D). The nucleoplasmic background between chromatin fibres contained little or no detectable material with a signature nitrogen to phosphorus ratio of RNPs [36], consistent with lower levels of transcription than seen at later stages.

We observed major changes in the nuclear landscape between pronuclei of the one-cell stage (Figure 2A–D) and nuclei of the two-cell stage (Figure 2E–H). The amount of chromatin associated with the nuclear envelope was increased. The nuclear perimeter was characterized by association with a thin rim of compact chromatin (arrowheads in Figure 2F,G,K). Also, a rim of compact chromatin varying from 30 to 60 nm in thickness “coats” the perimeter of the NPBs (arrowheads in Figure 2E,H, arrow in Figure 2M,N). In addition, chromatin in two-cell embryos was less uniformly dispersed and tended to organize into large compact domains compared to one-cell embryos (arrows in Figure 2K). 10 nm chromatin fibres could be observed within these domains, as well as on the periphery (arrowheads in Figure 2O), and occasionally in the nucleoplasm between these compact domains (arrow in Figure 2O). In both the one-cell and two-cell nuclei, the phosphorus content of the nucleoplasm was almost entirely derived from chromatin. Relatively few RNP structures could be detected between the chromatin fibres or blocks of compacted chromatin fibres, consistent with a relatively low level of transcription [42]. By the two-cell stage, the nuclear envelope and the surface of the NPBs have become competent for concentrating chromatin into these compartments.

### Highly dispersed chromatin is established by the 8-cell stage

**Figure 3:**
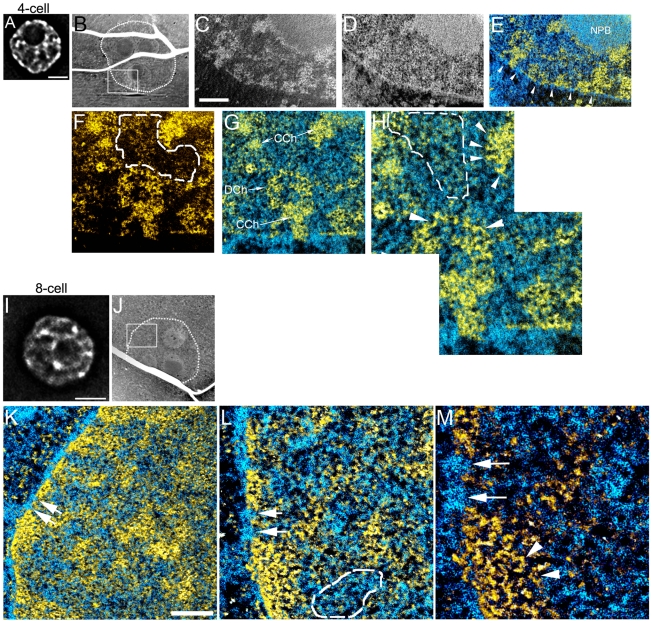
Chromatin becomes highly dispersed into a mesh of 10 nm fibres between four- and eight-cell stage embryos, with high levels of RNPs in the intervening nucleoplasmic space. (A), Fluorescence image of a DAPI stained nucleus of a four-cell embryo. (B), low magnification mass image of cell from four-cell embryo (nuclear periphery highlighted with dotted line). Field from boxed region (B) showing phosphorus and nitrogen maps (C,D), nitrogen map after phosphorus subtraction merged with phosphorus map (E). Nuclear pores are indicated (arrowheads, E), nucleolar precursor body (NPB). Higher magnification phosphorus map (F) and merged with nitrogen (G) of a field from region shown in C–E. DCh, dispersed chromatin; CCh, compact chromatin blocks. H, higher magnification of a field from region in F,G. Fields delineated with dotted lines (F,H) contain RNP structures, characterized by phosphorus-rich granules, but of a lower phosphorus to nitrogen ratio than chromatin fibres. Arrowheads (H) indicate 10 nm chromatin fibres. (I), Fluorescence image of a DAPI stained nucleus of an eight-cell embryo. (J), low magnification mass image of a cell from eight-cell embryo (nuclear periphery highlighted with dotted line). K, field shown in J of the merge of phosphorus and nitrogen signals. L, high magnification of field in K. Arrows serve as fiduciaries between K, L and M, and also indicate two nuclear pores. Area indicated with dotted lines shows high concentration of RNPs in nucleoplasm (L). M, high magnification of field in L. Arrowheads indicate 10 nm chromatin fibres in compact chromatin region at nuclear envelope. Scale bars in A and I represents 5 µm. Scale bar in C represents 500 nm (C–E), 250 nm (F,G) and 125 nm (H). Scale bar in K represents 500 nm (K), 250 nm (L) and 125 nm (M).

A dramatic change in nuclear architecture occurred between the two-cell and the eight-cell embryo, with the four-cell nuclei displaying an intermediate state (Figure 3). At the four-cell stage, both decondensed 10 nm nucleosomal chromatin fibres dispersed throughout the nucleoplasm (D Ch in Figure 3G,H) and blocks of more compact chromatin were prevalent, usually near the nuclear envelope but also in the nuclear interior (CCh in Figure 3C,G,H). As in the two-cell embryo, concentration of chromatin also occurred along the surfaces of the NPBs and at the nuclear envelope (Figure 3E). An accumulation of RNP structures in the nucleoplasm between the chromatin fibres are more prevalent in the four-cell nuclei than in nuclei of the two-cell stage. Phosphorus-rich RNP granules can be seen within the nucleoplasmic volume indicated by the broken line in Figure 3H.

The chromatin landscape of the eight-cell nuclei is easily distinguished from that of the two- and four-cell stages. The eight-cell nuclei had a more uniform distribution of chromatin fibres, with a less obvious delineation between the most compact domains and the dispersed chromatin between them. The small differences in chromatin density are obvious in the DAPI image (Figure 3I). The predominant chromatin fibre type was that of the 10 nm fibre, even in the most compact chromatin domains (arrowhead in Figure 3M), again indicating that DAPI alone appears to be a poor indicator of the degree of chromatin compaction. Besides the very dispersed chromatin architecture in nuclei at the eight-cell stage, the nucleoplasmic background was heavily populated with RNPs, nearly filling the space between the dispersed chromatin (area indicated in Figure 3L). The levels of RNPs in these nuclei far surpass those seen in either the two-cell or four-cell nuclei.

In summary, by the eight-cell stage, the chromatin has become highly dispersed, forming a mesh of 10 nm fibres throughout the nucleoplasm. The high concentration of RNPs between the chromatin fibres suggest that transcription levels appear to be elevated compared to previous developmental stages, which is consistent with previous RNA expression studies [43].

### Chromatin compaction levels differ between pluripotent and lineage-restricted cells

**Figure 4:**
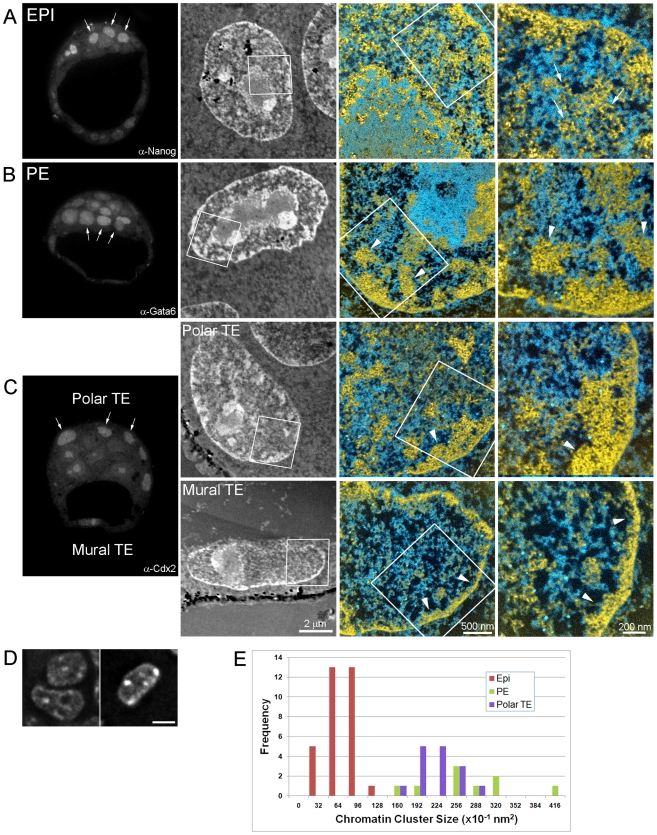
Chromatin is highly dispersed in pluripotent cell nuclei of E3.5 blastocysts, but more compact in lineage-committed cells. Left panels show fluorescence microscopy of physical sections (70 nm thickness) of E3.5 blastocysts immuno-labelled for Nanog (A, epiblast), Gata6 (B, primitive endoderm) and Cdx2 (C, polar and mural trophectoderm). Although auto-fluorescence was detected in the nucleolus of some cells, this does not impact the ability to distinguish between nuclei that are positive or negative for Cdx2/Gata6. Positive cells are indicated by arrows in each image. Panels in the second column show low magnification mass-sensitive image of a positive cell. Merged phosphorus and nitrogen maps of the area indicated in the mass-sensitive images are shown at two magnifications in the right-side panels. 10 nm chromatin fibres are prevalent in epiblast (arrows) whereas blocks of compact chromatin (arrowheads) with fewer dispersed 10 nm fibres are observed in extra-embryonic progenitor cells. (D), Fluorescence images of DAPI stained nuclei of epiblast (left) and trophoblast (right) cells of a E3.5 blastocyst (scale bar represents 5 µm). (E), Chromatin compaction was quantified by measuring the distribution of chromatin cluster size in each cell type (see materials and methodsfor details). PE and polar TE cells contain larger chromatin clusters than EPI cells, suggesting that lineage-restricted cells have a more condensed chromatin architecture than pluripotent cells.

Following the 8-cell stage, the first lineage decisions during development lead to the establishment of the embryonic and extra-embryonic tissues. These events provide an opportune model to compare chromatin structure between pluripotent and lineage-restricted cell types. We identified EPI, TE and PE cell types in E3.5 blastocysts by their expression of lineage-specific transcription factors using immunocytochemistry and then examined their chromatin structures using ESI (Figure 4). Pluripotent EPI cells (Nanog positive, arrows in Figure 4A left panel) displayed a well-dispersed 10 nm chromatin architecture (arrows in Figure 4A right panel) that is highly reminiscent of ES cells (Figure 4A, see results of stem cell lines section below) [11], [12]. In contrast, lineage-restricted PE cells (Gata6 positive, arrows in Figure 4B) and TE cells (Cdx2 positive, arrows in Figure 4C) revealed a more condensed chromatin architecture (arrowheads, Figure 4B,C). The higher compaction of chromatin in the lineage-committed cells is reflected in the differences in DAPI density in the epiblast vs. trophoblast cells (Figure 4D). Quantification of chromatin compaction levels was obtained by measuring the distribution of chromatin cluster sizes by ESI. This analysis revealed that TE and PE cells had larger domains of chromatin compared to EPI cells, thus confirming lineage-specific differences in compaction levels (Figure 4E).

We also observed differences within cells of the TE lineage that coincide with their developmental potential. The highly-proliferative polar TE cells contained blocks of compact chromatin of various dimensions along the nuclear envelope with connecting segments of compact chromatin that radiate into the nuclear interior (arrowheads in Polar TE, Figure 4C upper panel). In contrast, mural TE cells, which undergo endoreduplication to generate terminally differentiated giant cells, had a thin well-defined rim of compact chromatin along the nuclear envelope (approximately 100 nm thickness, arrowheads in Mural TE, Figure 4C, lower panel) and relatively few dispersed chromatin fibres throughout the nucleoplasm.

**Figure 5:**
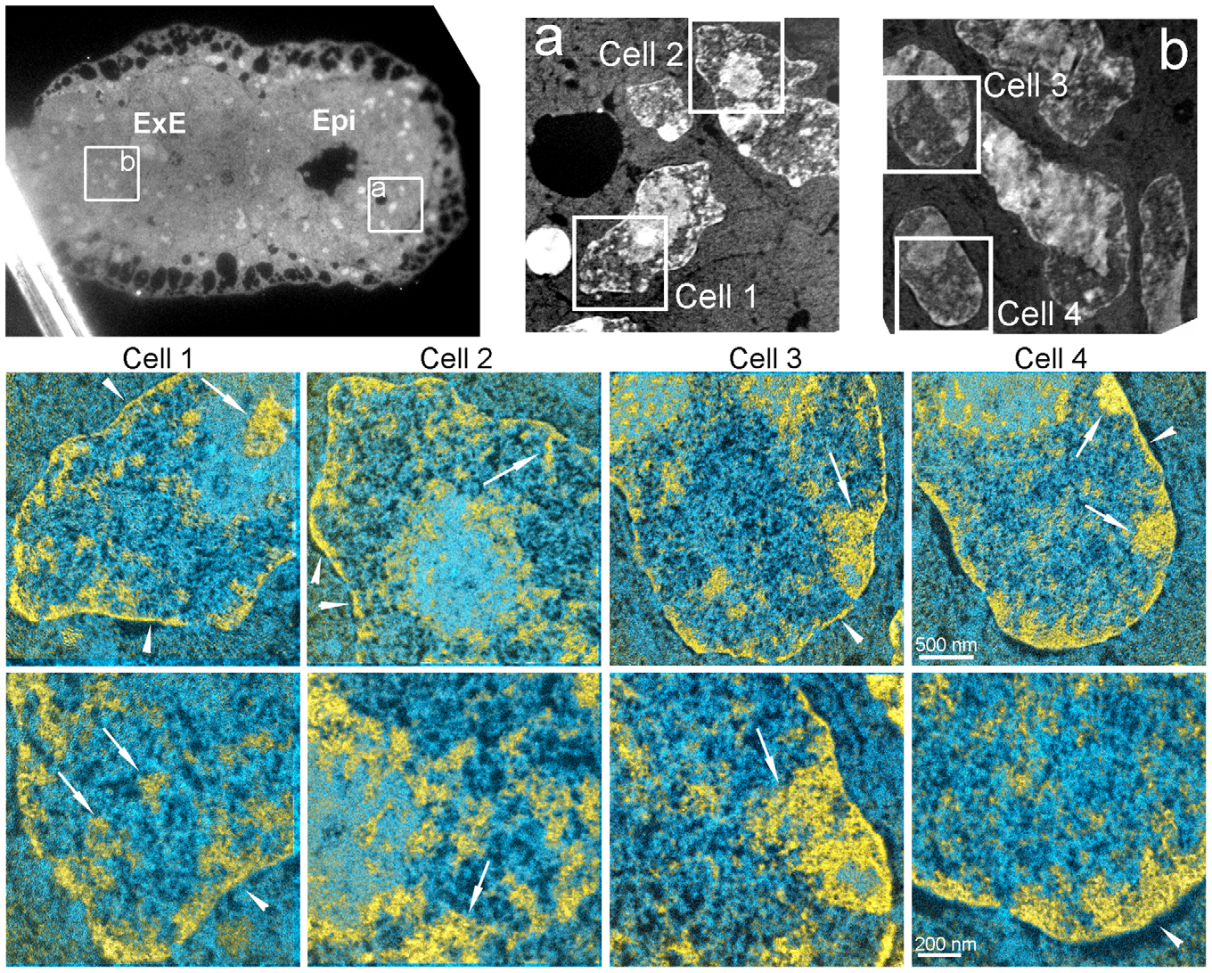
Chromatin forms compact domains along the nuclear envelope and throughout the nucleoplasm in embryonic and extra-embryonic progenitors at E5.5. Low magnification fluorescence micrograph (upper left) of E5.5 embryo, showing EPI and extra-embryonic ectoderm (ExE). Low magnification mass-sensitive image of (a) EPI and (b) ExE. Merged phosphorus and nitrogen maps of the indicated fields in (a) and (b) are shown at two magnifications. In both EPI and ExE, chromatin accumulates into compact rim along the nuclear envelope (arrowheads) and in compact blocks at the nuclear envelope and throughout the nucleoplasm (arrows) with little dispersed 10 nm chromatin visible between the compact chromatin domains.

Shortly after implantation, EPI cells undergo extensive epigenetic remodelling including random X-chromosome inactivation and *de novo* DNA methylation [44]. This transition is also associated with a restriction in developmental potential, as post-implantation stage EPI cells can no longer contribute to blastocyst chimeras [45], [46]. We, therefore, examined the chromatin architecture of EPI cells in early post-implantation stage (E5.5) embryos to investigate whether chromatin structure is also remodelled during these processes (Figure 5). EPI nuclei in these embryos were strikingly different from their counterparts in the blastocyst (Cells 1,2 in Figure 5). Chromatin was compacted into numerous blocks of widely varying shapes and sizes throughout the nucleus (arrows in Figure 5) and along the nuclear envelope (arrowheads in Figure 5). Uncompacted chromatin fibres between these blocks were sparse and 10 nm fibres were rare in and around the blocks of compact chromatin. The density of RNPs was also reduced relative to levels observed in preimplantation EPI cells or at earlier stages of development. Whereas EPI and TE cells were easily distinguished by ESI in E3.5 blastocysts (Figure 4A and C), nuclear and chromatin organization of E5.5 EPI and extra-embryonic ectoderm (ExE), which derives from TE, were essentially indistinguishable on the basis of chromatin and RNP distributions (Cells 3,4 in Figure 5).

From these studies, we conclude that a dispersed global chromatin architecture is characteristic of pluripotent cells and distinct from a more compact chromatin architecture formed in lineage-committed progenitor cells.

### Dispersed chromatin state in epiblast cells is dependent on pluripotency

We have shown that chromatin gradually decondenses during early development and establishes an open chromatin architecture in pluripotent EPI cells, which is very similar to ES cells (see results of stem cell lines section below) [11], [12]. We have also shown that lineage-restricted progenitor cells in the blastocyst have regions of compact chromatin. Together, these observations suggest that the establishment and maintenance of pluripotency may regulate, or be regulated by, chromatin structure. To begin to understand this inter-dependence, we examined the chromatin structure of EPI cells under conditions where pluripotency is not maintained in the embryo.

**Figure 6:**
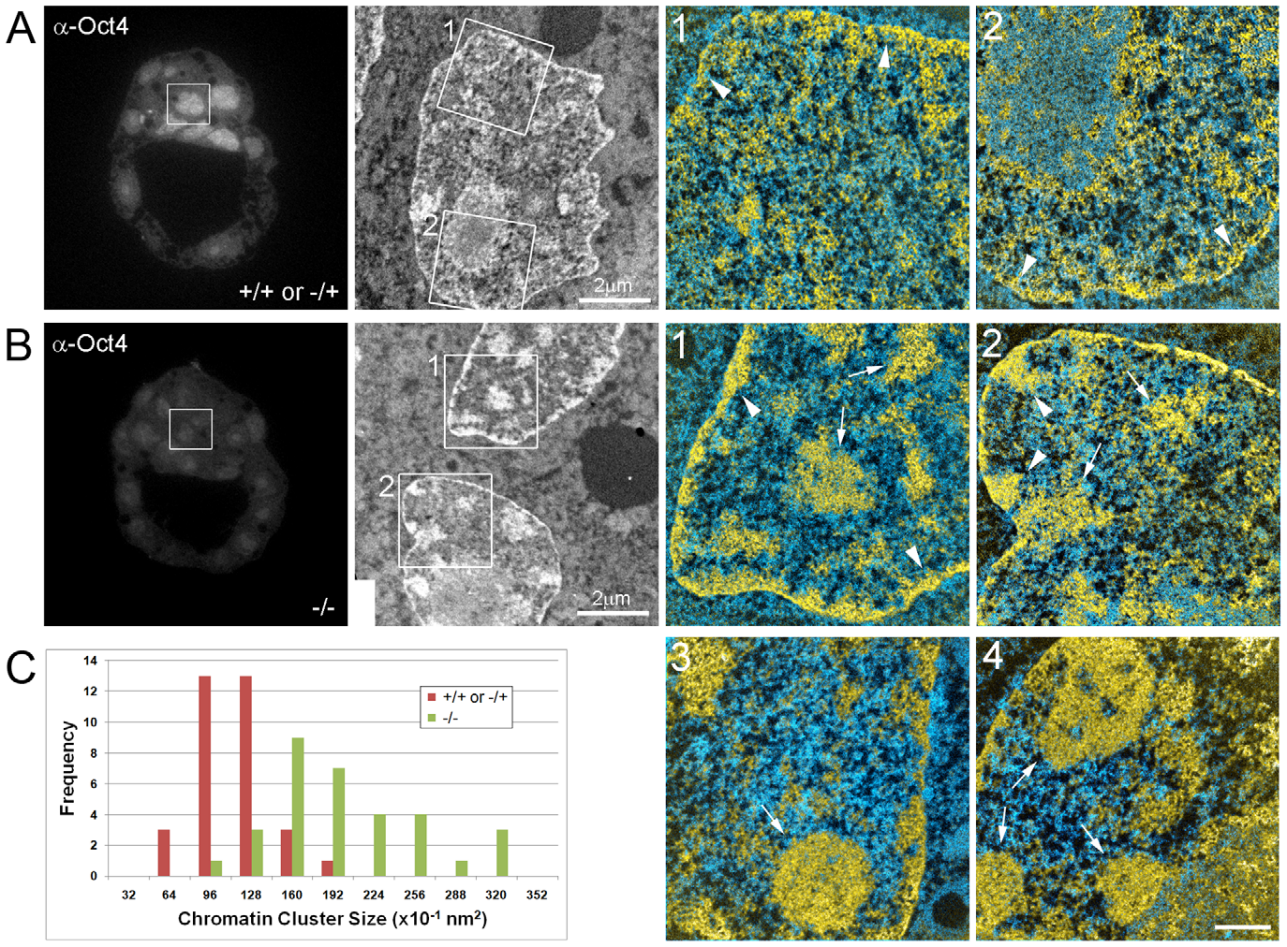
Loss of pluripotency results in chromatin compaction in epiblast cells. Left panels show fluorescence microscopy of Oct4 immuno-labelled embryos; wildtype Oct4^+/+^, heterozygous Oct4^−/+^ (A), and null mutants Oct4^−/−^ (B). Panels in the second column show low magnification mass-sensitive image of the area indicated in fluorescence images. Merged phosphorus and nitrogen maps are shown in the right panels. Panels labelled 3 and 4 are from a separate null embryo (not shown). Epiblast cells in control embryos have a thin rim of chromatin on nuclear membrane (arrowheads in A 1,2), whereas Oct4^−/−^ mutant epiblast cells showed greater accumulation of chromatin at the nuclear periphery (arrowheads in B 1,2). Arrows in B indicate large blocks of compact chromatin, a structure rarely observed in control epiblast nuclei. C, Chromatin compaction was quantified by measuring the distribution of chromatin cluster size in Oct4 mutant and control embryos (see materials and methodsfor details). Cells within Oct4^−/−^ embryos contained larger chromatin clusters than in control cells, suggesting that loss of Oct4 leads to increased chromatin compaction levels. Scale bar represents 500 nm in merged images.

Previous studies have shown that *Oct4*–null embryos initially establish morphologically normal blastocysts, with appropriate TE-restricted expression of Cdx2 in early blastocysts [47], [48]. However, late blastocyst stage mutants show ectopic expression of Cdx2 in putative EPI cells, and EPI cells lose their pluripotent status and are remodelled to a TE-like fate in the absence of Oct4 expression [47], [48]. We, therefore, examined the chromatin structure of EPI cells in early blastocysts that lacked zygotic expression of *Oct4*. We identified *Oct4*-null embryos by immunocytochemistry (Figure 6) and then imaged the nuclei of putative EPI cells by correlative fluorescence microscopy and ESI (Figure 6). EPI cells in control (*Oct4* ^+/+^ or *Oct4* ^−/+^, Figure 6A) embryos showed the expected dispersed chromatin structure, but in contrast, EPI cells from early blastocyst stage *Oct4* ^−/−^ embryos (Figure 6B) showed numerous regions of highly compacted chromatin (arrows in Figure 6B). Quantitative analysis confirmed these differences and revealed that the chromatin structure of mutant cells closely resembles the nuclei from TE cells (Figure 6C). Despite the altered chromatin structure in mutant early blastocysts, Cdx2 expression remained restricted to the TE and was not detected in EPI cells (data not shown), which is consistent with previous reports [48]. Thus, in conditions where pluripotency is not maintained in the embryo, extensive remodelling of chromatin architecture occurs and appears to precede the upregulation of TE transcription factor networks.

### Lineage-specific chromatin organisation is retained by their stem cell counterparts

**Figure 7:**
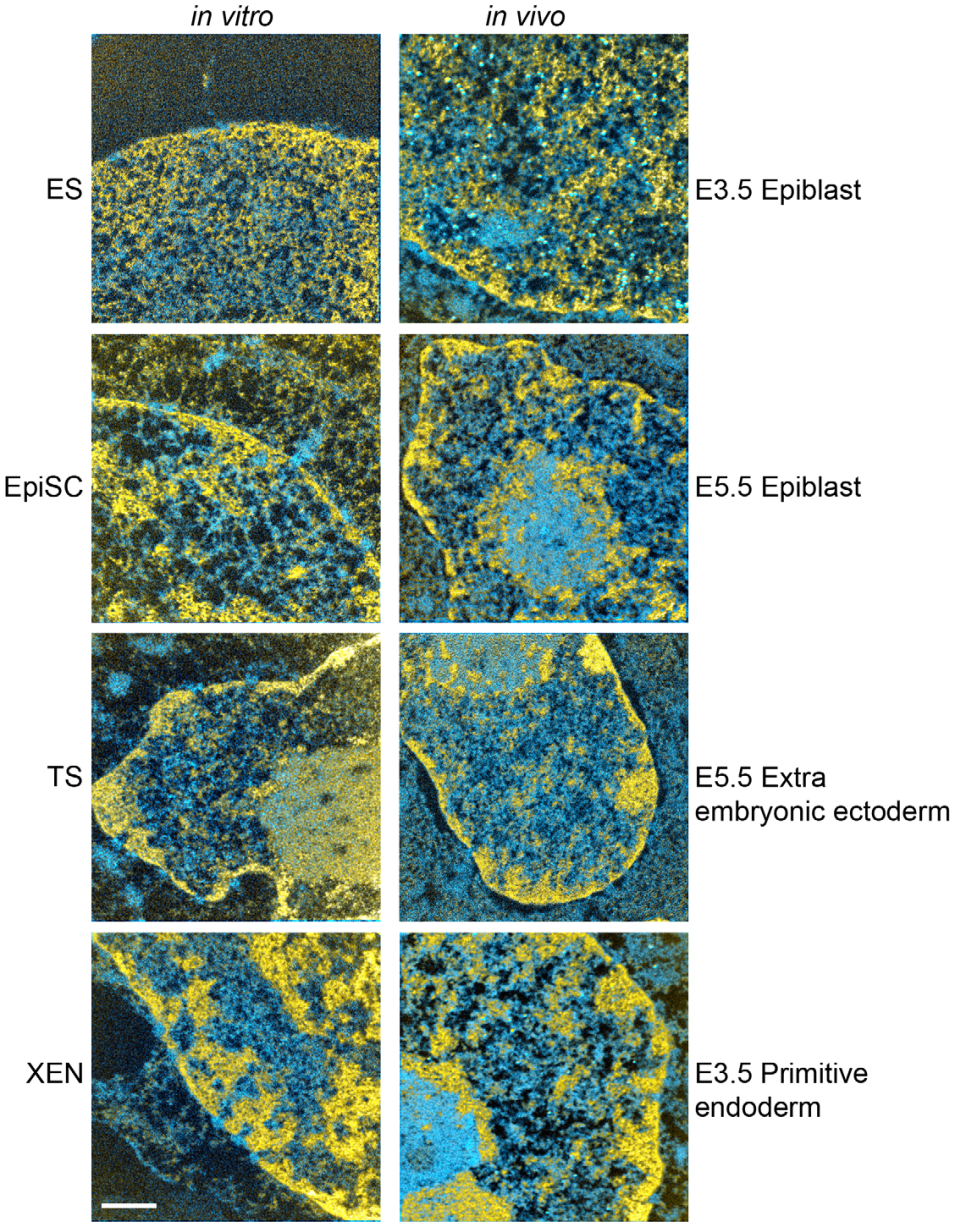
Global chromatin architecture in *in vitro* stem cell populations is similar to their *in vivo* counterparts in the embryo. ESI images of stem cells are shown in the left column (embryonic stem cells (ES), epiblast stem cells (EpiSCs), trophectoderm stem cells (TS), and extra-embryonic endoderm (XEN). ESI of nuclei of embryo stages from which the stem cells were derived are shown in the right column (E3.5 epiblast, E5.5 epiblast, E5.5 extra-embryonic ectoderm, E3.5 primitive endoderm). Chromatin (yellow) and protein and RNPs (shades of blue) are determined from nitrogen and phosphorus maps. Scale bar represents 500 nm.

In addition to pluripotent ES cells, other permanent stem cell lines that self-renew in culture can also be derived from pre- and post-implantation stage mouse embryos. These cell lines include trophoblast stem (TS) cells, which retain properties of ExE [49], extra-embryonic endoderm stem (XEN) cells, which retain properties of PE [50] and epiblast stem cells (EpiSCs), which retain properties of post-implantation EPI [51], [52]. The similarity in chromatin architecture between pre-implantation EPI and ES cells prompted us to compare these additional stem cell lines with their counterparts in the embryo. ESI revealed a striking similarity in chromatin organization between *in vivo* progenitors and their *in vitro* stem cell counterparts for all cell types examined (Figure 7). Thus, in addition to ES cells, these alternative stem cell lines will provide useful *in vitro* models to examine the lineage-specific regulation of chromatin structure.

## Discussion

**Figure 8:**
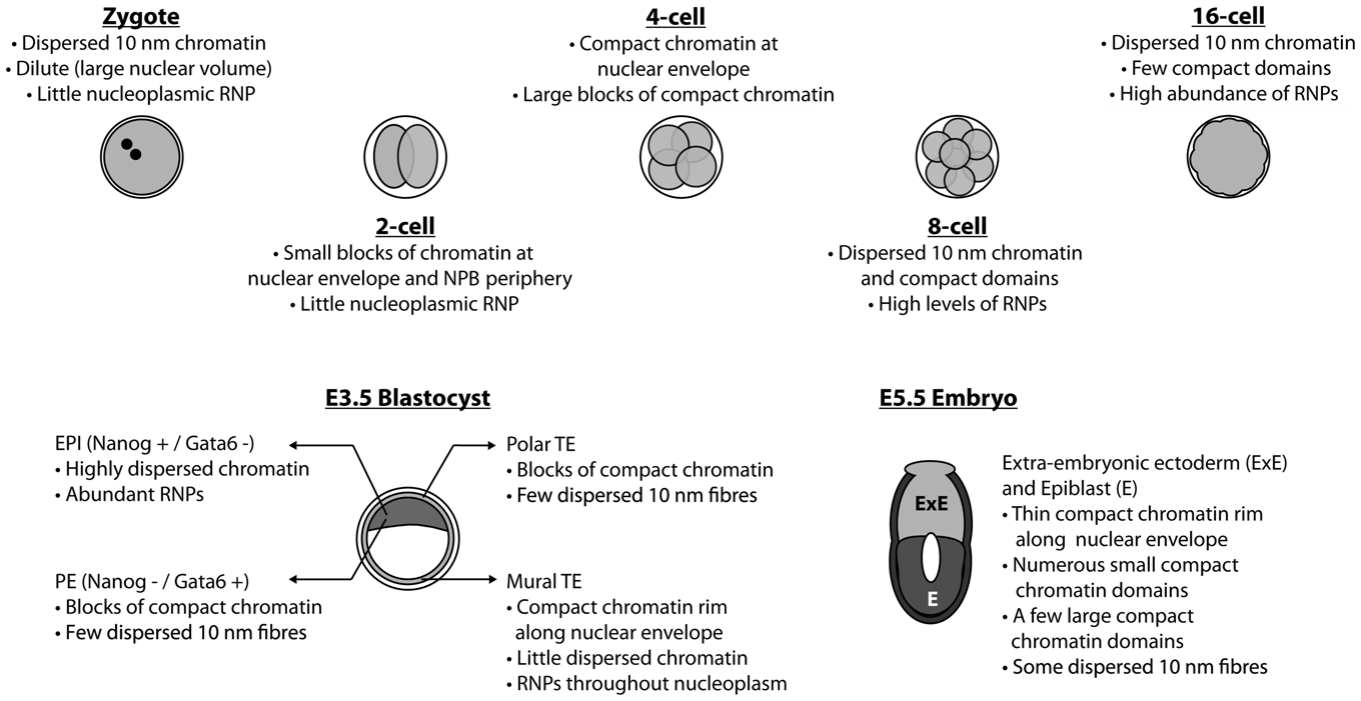
Major changes in nuclear and global chromatin structure occur in nuclei from the zygote to the E5.5 postimplantation stage embryo.

Using correlative light and electron spectroscopic imaging (ESI) to directly observe large-scale alterations or “remodelling” of global chromatin organization, we have found that dramatic changes in the chromatin landscape are associated with each stage from one-cell to postimplantation embryos (Figure 8).

In the pronuclei of the one-cell stage, the chromatin distribution is essentially featureless with an even and a dispersed distribution of 10 nm fibres. These fibres are highly contorted since only short segments can be followed in the thin 70 nm sections. A minimal degree of concentration of chromatin occurs at the surfaces of the NPBs and at the nuclear envelope. The necessary factors that act as adaptors between chromatin and the nuclear lamina [53] may not be present or have not been targeted to the nuclear envelope by this stage. Compartmentalization of chromatin into distinct structural and likely functional domains may not be required at the late one-cell stage since the genome is uniformly under-expressed, the transcript and protein requirements are supplied maternally. Low level transcription does occur, but the majority of these transcripts do not produce mature mRNA [54]. Thus, there may not be a requirement for functional compartmentalization of specific loci or chromatin domains at this stage.

Between the one-cell and two-cell stages, chromatin becomes structured and domains of compaction are prevalent. The nuclear envelope begins to concentrate significant amounts of chromatin, perhaps establishing a domain where gene silencing can be initiated. The appearance of compact chromatin at these stages is consistent with experiments that showed reduced transcriptional activity of episomal vectors between one-cell and two-cell stage embryos [55], [56]. In addition, we detected a very sparse distribution of RNPs throughout the nucleoplasm, which indicates that transcriptional levels are low at this stage in development. Together, these data suggest that the acquisition of a repressed epigenetic state precedes major zygotic transcriptional activity, which occurs in the mouse at the two-cell stage [57]–[59]. Besides organization at the nuclear envelope, the surfaces of the NPBs also take on a role in creating compartments. Indeed, α-satellite repeat DNA becomes targeted to these surfaces in the 2-cell nuclei [40] (and data not shown). The concentration of α-satellite chromatin contributed from all of the chromosomes to these sites is evidence for a non-random chromatin architecture. For this brief period in development, the surfaces of the NPBs may act as the functional equivalent of chromocentres found in differentiated mouse cells. Clustering of pericentric sequences into heterochromatic chromocentres may have regulatory potential by creating physical compartments for silencing.

At the eight-cell stage of development, we detected a dispersed and uniform chromatin architecture, suggesting that extensive epigenetic remodelling occurs after the two-cell stage. We also showed that this open chromatin structure, largely devoid of blocks of compact chromatin, is very similar to pluripotent EPI cells in the blastocyst. Fluorescence microscopy using the DNA counter-stain DAPI could be interpreted to reveal the presence of condensed chromatin domains at this stage of development [40], [41]. DAPI, however, is not a reliable determinant of DNA density. In contrast, the phosphorus signal derived from DNA by ESI is a quantitative measure of DNA density. DAPI can reveal differences in DNA compaction levels relative to the surrounding nucleoplasmic background, but this does not necessarily imply densely-packed chromatin domains [37]. Indeed, ESI indicates regions of differing chromatin density at the eight-cell stage, which would be contrasted by DAPI in fluorescence microscopy, but does not indicate the presence of compacted chromocentres. Importantly, this epigenetic state of relatively dispersed chromatin is also characteristic of undifferentiated ES cells, demonstrating that the distinct nuclear architecture is inherited from the embryo itself, rather than aberrantly acquired during stem cell derivation[11], [12] (Figure 7). Upon ES cell differentiation, chromatin compaction occurs by forming distinct chromocentres and also increased levels of compaction at the nuclear envelope [11]. Similarly, lineage-restriction in the embryo is also associated with altered chromatin structure. TE and PE cells of the blastocyst show blocks of chromatin compaction radiating away from the nuclear envelope into the nuclear interior. This may represent the first substantial and permanent sub-nuclear domain to serve as a gene silencing environment [53].We conclude, therefore, that a uniformly dispersed chromatin configuration is a hallmark of the undifferentiated and pluripotent state.

How might the dispersed chromatin state be established during development? Loss of function studies in mice and ES cells have revealed that chromatin remodelling proteins are required for the establishment of EPI cells and also for the maintenance of ES cells [14]–[19]. However, interactions between epigenetic mediators and the core transcriptional circuitry in pluripotent cells remain poorly defined. Genome-wide association studies in ES cells show that Oct4 and Nanog bind to the promoters of multiple chromatin remodelling factors and may be required to ensure their continued expression [60], [61]. In particular, *Nanog* levels are critical for X-chromosome inactivation during ES cell differentiation [62] and so it will be important to determine whether the regulation of other pluripotency-associated epigenetic processes, such as chromatin compaction, may be similarly controlled.

Together, our studies show that global chromatin organisation may provide the structural basis in the nucleus that distinguishes pluripotent cells from tissue-restricted progenitor cells.

## Materials and Methods

### Embryo collection

Three and a half week-old female mice (C57Bl6 from The Jackson Laboratory, Bar Harbor, Maine, USA) were super-ovulated by intraperitoneal injection of 5 IU pregnant mare's serum gonadotropin (Calbiochem, La Jolla, California USA) followed 46 h later by 5 IU human chorionic gonadotropin (hCG; Sigma-Aldrich, St Louis, MO, USA). Embryos at different stages of preimplantation development were flushed from oviducts or uterine horns with M2 medium (Sigma-Aldrich, St Louis, MO, USA) as previously described [63]. *Oct4*-null embryos were generated by breeding Pou5f1^tm1Scho^ heterozygous mice [64]. Blastocyst and postimplantation stage embryos (ICR strain; not super-ovulated) were isolated at embryonic days E3.5 and E5.5, respectively, in DMEM/F12 (Invitrogen, Burlington, ON, Canada) supplemented with 10% fetal bovine serum (Wisent, St-Bruno, QC, Canada). Reichert's membranes were removed using 30-gauge needles. All embryos were fixed immediately after collection and processed as described in the next sections.

The use of mice in this study was approved by the Animal Care Committee of The Hospital for Sick Children, Toronto, Ontario, Canada. In accordance with the “Animals for Research Act of Ontario” and the Guidelines of the Canadian Council on Animal Care, mice were provided with the healthiest conditions and most humane care practically possible. Mice were maintained in the animal facility at normal temperature (21–23°C) and 12 hour light/dark cycle with free access to water and food.

### Immunofluorescence Labeling

Embryos were washed in phosphate buffered saline (PBS) and fixed in fresh 4% paraformaldehyde (Electron Microscopy Sciences (EMS); Fort Washington, PA, USA) in PBS (pH 7.5) for 30 min at room temperature (RT). Fixed embryos were washed three times with PBS and permeabilized in PBS containing 0.5% Triton X-100 for 10 min at RT, and rinsed twice with PBS. Permeabilized embryos were labeled with mouse anti-Cdx2 (1∶200, Biogenex CDX2-88), rabbit anti-Cdx2 (1∶10) [65], mouse anti-Oct4 (1∶100, Santa Cruz C-10), rabbit anti-Nanog (1∶200, ReproCell) and goat anti-Gata6 (1∶200, R&D) for 2 hours at RT. The secondary antibody labeling was carried out with one of the fluorescently labeled antibodies of Cy3- or Cy5-labelled donkey anti-mouse (Jackson ImmunoResearch Laboratories, Inc. West Grove, PA, USA), Alexa488 or Alexa546-conjugated anti-mouse, rabbit or goat IgG (Molecular Probes). Immuno-labeled embryos were processed for electron spectroscopic imaging.

### Correlative light and electron spectroscopic imaging

Following immuno-labelling, the embryos were postfixed in 2% glutaraldehyde (EMS) in PBS for 10 minutes. They were washed three times with PBS and three times with double distilled water (Invitrogen Canada Inc., Burlington, Ontario, Canada). After dehydration in a series of graded ethanol steps of 30, 50, 70, and 90%, with incubations for 2 hours at each step, and overnight incubation in 100% ethanol at 4°C, dehydrated embryos were embedded in Quetol 651 resin (EMS) as previously described [66]. Serial sections of 70 nm thickness were obtained by an Ultracut UCT ultramicrotome (Leica Microsystems Inc., Bannockburn, IL, USA), and were picked up onto finder grids. These grids allowed fluorescence imaging of particular embryonic cells in a physical section, and then easily finding them later in the electron microscope. Electron micrographs were obtained at 200 kV on a transmission electron microscope (Tecnai 20, FEI, Hillsboro, Oregon, USA). Energy filtered images were collected using a post-column imaging filter (Gatan Inc., Warrendale, PA, USA) as described elsewhere [67]. Low magnification, mass-sensitive images are recorded where the total field includes an entire cell or a few cells in close proximity. These are used to correlate with images recorded of the physical section with the fluorescence microscope. Elemental maps were generated by dividing the element-enhanced post-edge image by the pre-edge image. Net ratio elemental maps were derived from pre- and post-edge images recorded at 120 and 155 eV for phosphorus, and at 385 and 415 eV for nitrogen. In presenting the phosphorus and nitrogen signals, the phosphorus signal is shown in shades of yellow. The phosphorus map is also subtracted from the nitrogen map, with a normalization factor between the two maps that confers zero signal in chromatin-based structures. Nitrogen above this signal is shown in shades of cyan. Hence, protein-based structures appear as blue, and RNP (ribonucleoprotein) structures, which have a lower phosphorus to nitrogen ratio than chromatin, appear in intermediate shades of yellow and cyan in the merged images. Because the nitrogen signal is normalized to zero under the chromatin fibres, chromatin appears in shades of only yellow in the merged images. The images were processed using Digital Micrograph (Gatan) and Adobe Photoshop CS version 8.0 (Adobe Systems Incorporated). DAPI images from the same stage embryos were obtained with inverted Olympus 1X81 microscope and were deconvolved with Image-Pro Plus 6.2 (Media Cybernetics, Inc. Bethesda, MD, USA).

Quantification of chromatin compaction levels was achieved as follows. Net phosphorus images were segmented to eliminate the phosphorus signal contributed by RNPs, which have a significantly higher N:P ratio than chromatin, and the resulting phosphorus signal was binarized. ImageJ was then used to find clusters of phosphorus signal consisting of 4 or more pixels (Analyze Particles tool). This software tool finds hundreds of pixel clusters ranging in size from 4 pixels to thousands of pixels, depending on the degree of chromatin compaction. The average pixel cluster size is provided and this value was used to create histograms for each cell type analyzed. Cells with more compact chromatin were characterized by having larger clusters of chromatin. For each cell type, between 20–30 fields of view from serial sections of approximately 10 cells were analyzed. Fields of view for analysis were selected that did not contain regions from the nucleolus of the cells. The minimum number of clusters in any analyzed field was approximately 300.

In the structural analysis, we examined a minimum of three embryos from at least two separate experiments for each developmental stage. Multiple serial sections were imaged so that the chromatin architecture of sections through the nuclear periphery would not be confused with that of the nuclear interior. To avoid potential differences in chromatin architecture related to cells entering or exiting mitosis at the early stages (e.g. one, two and four-cell), embryos were harvested at intervals well separated temporally from mitosis [40]. At later stages, cells are no longer synchronous, but the chromatin morphology of cells entering prophase or exiting telophase is easily recognized by DAPI in the fluorescence microscope and by ESI, and such cells were excluded in the analysis.
